# Debate: Urban–rural environments – which is better for mental health? Moving beyond urban–rural dichotomies in psychosis risk for young people

**DOI:** 10.1111/camh.12761

**Published:** 2025-03-21

**Authors:** James B. Kirkbride

**Affiliations:** ^1^ PsyLife Research Group, Division of Psychiatry UCL London UK

**Keywords:** Psychotic disorders, urban health, social determinants of health, social cohesion, epidemiology

## Abstract

While urban–rural gradients exist for common mental disorders (Wiers et al., 2025), observations from the Global North suggest these are strongest for psychotic disorders, which typically emerge during adolescence. Despite well‐documented urban–rural variation in risk, recent research suggests a more nuanced relationship between place and these severe mental illnesses exists. Traditional urban–rural dichotomies cannot account for social gradients in psychosis globally for young people. Instead, a framework centred on social identity, inclusion and belonging may provide a more comprehensive approach to understanding psychosis risk as a result of the environments in which people are born, raised and live. Future research should integrate traditional epidemiological designs with causal inference methods and new technologies to capture momentary responses to diverse environmental stimuli that are both place‐based and placeless. This approach could uncover novel avenues for prevention and intervention, tailored to the digital age, ultimately improving outcomes for young people and families affected by psychosis.

In their debate piece on whether urban or rural environments are better for the mental health of young people, Wiers et al. (2025) focus on the link between urban upbringing, well‐being and common mental disorders. These are particularly prescient concerns given both a rapidly urbanising global population and rapidly rising incidence rates of these conditions among adolescents (Dykxhoorn et al., 2024). Their debate rightly highlights the importance of selection into urban environments as a non‐causal explanation of urban–rural variation in rates, the potentially causal role of urban environments as habitats of stress sensitisation, and the need to examine the physical and/or social environmental conditions that differentiate urban and rural environments, which may account for variation in risk.

Clues to inform this debate can be found in the literature on the epidemiology of psychosis, where these same issues have been well rehearsed for at least a century (Faris & Dunham, 1939). Psychosis, characterised by a loss of contact with reality, typically first emerges in adolescence with a peak onset in schizophrenia spectrum disorders (SSD) of around 20 years old (Solmi et al., 2022). These disorders have been shown to have a strong urban–rural gradient in many countries in the Global North. This was first demonstrated by the seminal work of two sociologists in Chicago, Faris and Dunham (1939), who investigated urban gradients in the hospitalised incidence of various mental disorders throughout the 1920s across the distinctly configured census tracts of the Windy City during a period of enormous social transition in both the city itself and across the United States of America. They found that schizophrenia, but not bipolar disorder, displayed remarkably strong centripetal patterning, with highest rates occurring in the most densely populated inner‐city census tracts characterised by high levels of social instability and poverty, in a pattern observed in many other Western European cities thereafter (March et al., 2008).

Urban–rural gradients in psychosis risk have subsequently been extended in longitudinal studies of urban birth and upbringing (March et al., 2008), with individuals born and raised in urban environments demonstrating higher rates of psychotic disorders compared with their rural counterparts, lending some support for a potential etiological role. Identifying these social and spatial gradients is crucial for several reasons. First, our current methods for primary prevention of psychosis (like many mental health conditions) are woefully inadequate (Kirkbride et al., 2024), so identifying potentially ameliorable targets for intervention in increasingly urban populations globally underscores this as a legitimate concern for public mental health. Second, understanding urban–rural gradients in mental health can inform secondary prevention strategies, potentially allowing for earlier intervention that leads to improved outcomes for those at risk.

Scholars, including the present author, have long been captivated by the alluring simplicity of understanding variation in the occurrence of psychosis – a condition of altered percept – as a direct function of living in environments that demand heightened sensory and cognitive navigation and adaptation. Urban environments, although infinite in form, function, culture and structure, are reservoirs of positive, negative and neutral external stimuli that demand innate attention and processing. Within these urban contexts, those that threaten – often those that objectively or subjectively lack material, social or cultural capital to permit psychologically robust habitus adaptation – may create conditions that engender psychosis. But despite the appeal of such an explanation, there are several reasons to caution against aligning gradients in psychosis risk to simple urban–rural dichotomies, or indeed any other unidimensional construct.

One notable limitation in this regard is that urban–rural gradients observed for SSDs do not extend to affective psychotic disorders (Faris & Dunham, 1939), suggesting a more nuanced relationship between place and psychosis subtypes exists. Second, urban–rural gradients in SSD risk could be attributable to the related concepts of gene–environment correlation (*r*GE) and reverse causality. The aforementioned longitudinal association between urban birth and later SSDs refutes reverse causality – that is, selection of people with psychosis into more urban environments – as an explanation of higher rates in more urban environments. This evidence also partially argues against *active r*GE (where an individual seeks out environments that complement their own genetic predisposition to psychosis). *Passive r*GE, however, whereby parents provide both genetic and environmental exposures to their children, remains a possible explanation for higher rates of SSDs in more urban environments. This situation potentially arises because of considerable genetic and phenotypic overlap between cognition and psychosis (Knowles et al., 2021), which means that parents (and their ancestors) at higher genetic risk of psychosis may also be more likely to reside in more adverse social environments over successive generations as a result of subtle but influential impacts on cognition that limit educational, occupational and economic opportunities. While evidence for passive *r*GE between genetic risk for schizophrenia and urbanicity exists, it may not confound associations between urban birth and later psychosis risk (Solmi, Lewis, Zammit, & Kirkbride, 2020).

Third, traditional epidemiological studies that have investigated urban–rural and other place‐based variation in psychosis risk are geographically rooted in two assumptions: first, that residential address is a proxy for all environmental exposure; and second, that urban–rural gradients in mental health found in the Global North would apply to all cities globally. There is good reason to question both axioms. For example, evidence from the Global South shows that urban–rural gradients in psychosis risk do not apply universally across all countries (DeVylder et al., 2018), while technology now allows us to gather real‐time sensory feedback to understand effects on young people's mental health as they move through different environments in their daily lives (Russell & Gajos, 2020).

The above challenges suggest that the urban–rural dichotomy may not be the most relevant framework for understanding social gradients in youth mental health outcomes, including psychosis. By reframing this evidence, we see that urban gradients in schizophrenia are most strongly associated with greater material deprivation and social fragmentation (Faris & Dunham, 1939). These social conditions, along with the role of the physical environment (pollution, access to green space and visual identity), give neighbourhoods power to act as reservoirs of both risk and resilience (March et al., 2008) that shape their inhabitants identity, behaviour, cognition and perception. Seen through this lens, observed social and spatial gradients in psychosis risk coalesce around themes of social belongingness, inclusion and psychosocial (dis)empowerment. This potentially allows us to develop a strong, intersectional framework for understanding the aetiology of psychosis and rectifying diverse observations from different studies.

Concepts such as ethnic density, social capital and isolation all have mechanisms that operate through social belongingness, inclusion and psychosocial (dis)empowerment. The ethnic density effect, where lower rates of psychotic disorders are observed for some minoritised ethnic groups when living in areas with a higher proportion of people with similar ethnic identities, is consistent with a role for social connection and cultural congruency in the aetiology of psychosis. In related research, the elevated risk of psychotic disorders experienced by several minoritised ethnic groups has been shown to be a product of greater sociocultural distance from the majority population (Jongsma et al., 2021), underscoring how social belongingness may buffer effects of discrimination, socio‐economic position or systemic barriers to good mental health. Similarly, higher levels of social capital, characterised by trust, reciprocity and civic engagement within communities, have been associated with lower rates of psychotic disorders (March et al., 2008). This more nuanced conceptualisation of place and belonging tracks with common mental disorders as well. For example, research in a cohort of Canadian youth has demonstrated that strong longitudinal associations between stressful life events and several internalising and externalising disorders were only apparent for teenagers growing up in neighbourhoods with low social cohesion (Kingsbury, Clayborne, Colman, & Kirkbride, 2020).

Moving beyond simple urban–rural dichotomies to understand psychosis and other mental health conditions in contemporary cohorts of children and young people will also require us to reframe risk and resilience as both place‐based and placeless, especially in our increasingly digitally connected (and potentially digitally excluded) societies. In the context of young people's lives, social environments encompass not only traditional physical locations like home, school, university or work, but virtual spaces and online communities.

The shift away from a simplistic urban–rural dichotomy towards a more comprehensive understanding of place‐based and placeless risk and resilience factors has important implications for future research and intervention strategies. Longitudinal studies that investigate the role of communities of belonging, whether aligned to physical or virtual environments, will be needed to elucidate the complex interplay between place, social connection and psychosis risk in young people. Integration of traditional epidemiological study design with technologies that allow the capture of momentary responses to these broad, diverse environmental stimuli (Russell & Gajos, 2020) could uncover new avenues for primary prevention and intervention that go beyond traditional place‐based strategies, tailored to young people most at risk of common and severe mental disorders.

## Funding information

This work was supported by UK Research and Innovation funding for the Population Mental Health consortium (grant no MR/Y030788/1) which is part of Population Health Improvement UK (PHI‐UK), a national research network that works to transform health and reduce inequalities through change at the population level.

## Conflict of interest statement

The authors have declared that they have no competing or potential conflicts of interest.

## Ethics statement

No ethical approval was required for this debate article.

## Data Availability

Data sharing is not applicable to this article as no new data were created or analysed.
